# Biotechnological Utilization of Amazonian Fruit: Development of Active Nanocomposites from Bacterial Cellulose and Silver Nanoparticles Based on *Astrocaryum aculeatum* (Tucumã) Extract

**DOI:** 10.3390/ph18060799

**Published:** 2025-05-26

**Authors:** Sidney S. dos Santos, Miguel Ângelo Cerqueira, Ana Gabriela Azevedo, Lorenzo M. Pastrana, Fauze Ahmad Aouada, Fabrício C. Tanaka, Gustavo Frigi Perotti, Marcia Regina de Moura

**Affiliations:** 1 Hybrid Composites and Nanocomposites Group (GCNH), Department of Physics and Chemistry, Ilha Solteira School of Engineering, São Paulo State University (UNESP), Ilha Solteira CEP 15385-000, SP, Brazil; fauze.aouada@unesp.br (F.A.A.); marcia.aouada@unesp.br (M.R.d.M.); 2 Instituto de Ciências Exatas e Tecnologia, Universidade Federal do Amazonas (UFAM), Itacoatiara CEP 69104-404, AM, Brazil; gustavoperotti@ufam.edu.br; 3 International Iberian Nanotechnology Laboratory, Av. Mestre Jos’e Veiga, 4715-330 Braga, Portugal; miguel.cerqueira@inl.int (M.Â.C.); ana.azevedo@inl.int (A.G.A.); lorenzo.pastrana@inl.int (L.M.P.); 4 Department of Food Engineering, Faculty of Animal Science and Food Engineering, USP—University of São Paulo, Av. Duque de Caxias Norte, 225, Pirassununga CEP 13635-900, SP, Brazil; tanaka.fabricio@usp.br

**Keywords:** bacterial cellulose, silver nanoparticles, green chemistry

## Abstract

**Background/Objectives:** The rise of bacterial resistance and the search for alternative, biocompatible antimicrobial materials have driven interest in natural-based nanocomposites. In this context, silver nanoparticles (AgNPs) have shown broad-spectrum antibacterial activity, and bacterial cellulose (BC) is widely recognized for its high purity, hydrophilicity, and biocompatibility. This study aimed to develop a bio-based BC–AgNP nanocomposite via green synthesis using *Astrocaryum aculeatum* (tucumã) extract and assess its antimicrobial performance for wound dressing applications. **Methods:** BC was biosynthesized via green tea fermentation (20 g/L tea and 100 g/L sugar) and purified prior to use. AgNPs were obtained by reacting aqueous tucumã extract with silver nitrate (0.1 mmol/L) at pH (9) and temperature (40 °C). BC membranes were immersed in the AgNPs dispersion for 7 days to form the nanocomposite. Characterization was performed using UV–Vis, DLS, TEM, SEM–EDS, FTIR, XRD, ICP–OES, and swelling analysis. Antibacterial activity was evaluated using the disk diffusion method against *Staphylococcus aureus* and *Escherichia coli* (ATCC 6538 and 4388). **Results:** The UV–Vis spectra revealed a gradual decrease in the surface plasmon resonance (SPR) band over 7 days of incubation with BC, indicating progressive incorporation of AgNPs into the membrane. ICP analysis confirmed silver incorporation in the BC membrane at 0.00215 mg/mL, corresponding to 15.5% of the initial silver content. Antimicrobial assays showed inhibition zones of 6.5 ± 0.5 mm for *S. aureus* and 4.3 ± 0.3 mm for *E. coli*. **Conclusions:** These findings validate the successful formation and antimicrobial performance of the BC–AgNP nanocomposite, supporting its potential use in wound care applications.

## 1. Introduction

While microbial resistance is a natural evolutionary process, its acceleration has been largely driven by the indiscriminate use of antibiotics [1]. The indiscriminate use of antibiotics has made bacterial resistance one of the primary public health concerns today [2,3,4]. This phenomenon leads to clinical and economic impacts, including increased mortality rates, prolonged hospital stays, and higher treatment costs, potentially overwhelming public healthcare systems in a short time [2,3,4]. Therefore, new therapeutic approaches are being explored to address this issue [5,6].

Some natural hydrophilic polymers can absorb and retain a substantial amount of water in their structure, forming a three-dimensional network. They are called hydrogels. Such hydrogels are promising for wound dressing applications, particularly for burn treatment, due to their ability to maintain a moist environment that promotes tissue regeneration. Unlike plant cellulose, bacterial cellulose (BC) exhibits higher crystallinity, greater hydrophilic capacity, and better mechanical strength in contact with water [4,7]. BC is a natural polymer produced by a group of Gram-negative bacteria from the following genera: *Acetobacter*, *Achromobacter*, *Aerobacter*, *Agrobacterium*, *Alcaligenes*, *Azotobacter*, *Escherichia*, *Komagataeibacter*, *Pseudomonas*, *Rhizobium*, and *Sarcina* [8]. A cost-effective and efficient approach to obtaining BC is associated with the production of a fermented beverage from sugary teas and a symbiotic culture of bacteria and yeast, commonly known as SCOBY [9,10].

To enhance the biomedical performance of BC, several studies have focused on its functionalization with antimicrobial agents, especially silver nanoparticles (AgNPs), known for their broad-spectrum bactericidal activity [6,11]. Silver nanoparticles (AgNPs) have attracted great attention due to their high inhibitory effect on pathogens [10,12]. Among the various synthesis methods, the use of plant extracts represents an environmentally sustainable approach [13] in which plant biomolecules and secondary metabolites, such as amino acids, proteins, and polysaccharides, reduce silver ions into metallic silver, a redox reaction. Then, the biomolecules adhere to the surface of the AgNPs, aiding in the stabilization of the produced colloids [14,15].

Plant extracts are used in many industrial and scientific sectors, such as pharmaceutical, perfume, handicraft, and food production industries [16,17], driving many studies in the search for plant bioactives [18]. The Amazon rainforest has the highest plant diversity worldwide; however, this great diversity has limited technological exploitation [19]. The tucumã (*Astrocaryum aculeatum*) is an endemic fruit of the Amazon region, whose commercialization is concentrated in the city of Manaus, Amazonas, and the surrounding areas. It is consumed fresh, in the form of sandwiches, ice cream, pizzas, and traditional regional dishes. Recent studies have shown that the fruit has a high concentration of bioactive compounds, with phytochemical constituents potentially involved in the green synthesis of silver nanoparticles (AgNPs) [20,21,22].

Despite its nutritional and economic value, *A. aculeatum* remains underexplored from a phytochemical and technological perspective, especially regarding its potential in nanomaterial synthesis [22]. The fruit presents a rich composition of phytochemicals with potential involvement in the synthesis of AgNPs, notably the significant presence of unsaturated fatty acids such as 11-octadecenoic acid and linoleic acid, as well as glycerides like 1,3-diolein, glycerol 1-oleate 3-stearate, 1-monoolein, and 1-palmitoyl-3-oleoylglycerol. Palmitic acid, representing saturated fatty acids, and cycloartenol, a triterpene, also stand out. These compounds possess functional groups capable of acting as both reducing and stabilizing agents during the synthesis of AgNPs, contributing to the reduction of silver ions and the colloidal stabilization of the formed nanoparticles [23].

Due to its significant bactericidal potential, silver has been used since ancient times as a tool for treating various infections [24]. Popescu et al. [25] investigated the wound healing effect of chitosan-based hydrogels with AgNPs incorporated on their surface. The nanocomposite exhibited high healing potential, indicating its promise as a wound dressing. Recent research suggests that AgNPs have great potential in treating and healing wounds [26,27]. The incorporation of these nanomaterials into natural polymeric surfaces, such as BC, has attracted great attention. Because AgNPs have high affinity and adhesiveness to biological membranes, they can be used in many applications, for example, the BC–AgNP nanocomposite can be used to treat burns [28,29]. The combination of these materials synergistically enhances both antimicrobial and regenerative properties, contributing to accelerated wound healing. Zhou et al. [30] analyzed the antimicrobial activity and cytotoxicity of AgNPs inserted into cellulose nanofibers. Cytotoxicity assessment demonstrated a growth rate > 75% and was effective against *Staphylococcus aureus*, *Escherichia coli*, and *Listeria monocytogenes*.

Considering the promising characteristics of both bacterial cellulose and silver nanoparticles, and the unique phytochemical profile of tucumã extract, their combination represents a sustainable strategy for producing bioactive nanocomposites. In this study, AgNPs were produced through green synthesis using the aqueous plant extract of the Amazonian fruit *A. aculeatum* and then incorporated into BC through immersion. The formation and properties of the BC–AgNP nanocomposite were monitored and characterized. The amount of silver in the AgNPs dispersion and in the BC-AgNP nanocomposite was also investigated using inductively coupled plasma (ICP) analysis. Finally, the antimicrobial effect of the BC–AgNP nanocomposite as a potential wound dressing was tested against Gram-positive (*S. aureus*) and Gram-negative (*E. coli*) bacteria.

## 2. Results

### 2.1. Production and Characterization of AgNPs

**Figure 1:**
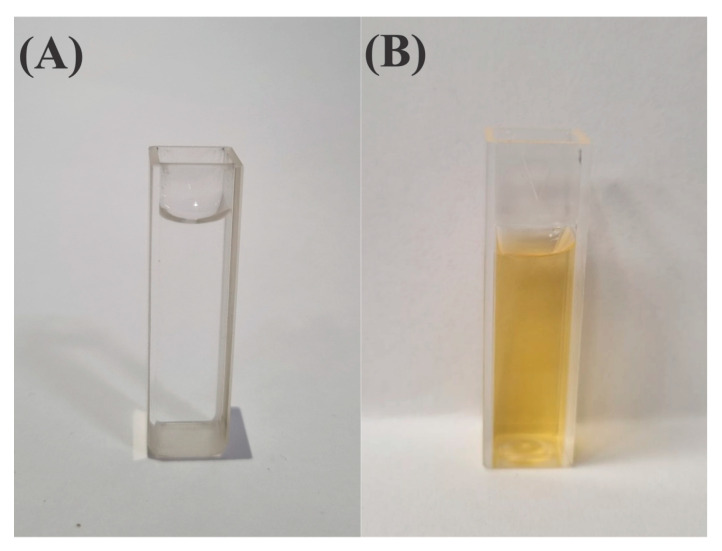
AgNPs dispersion at (**A**) time zero and (**B**) after 3 days of reaction.

Following the principles of green chemistry, AgNPs were synthesized using biomolecules from the plant extract of an Amazonian plant, commonly known as tucumã, as reducing and stabilizing agents [31]. Dispersed AgNPs can be observed in Figure 1B, 3 days after preparation.

**Figure 2:**
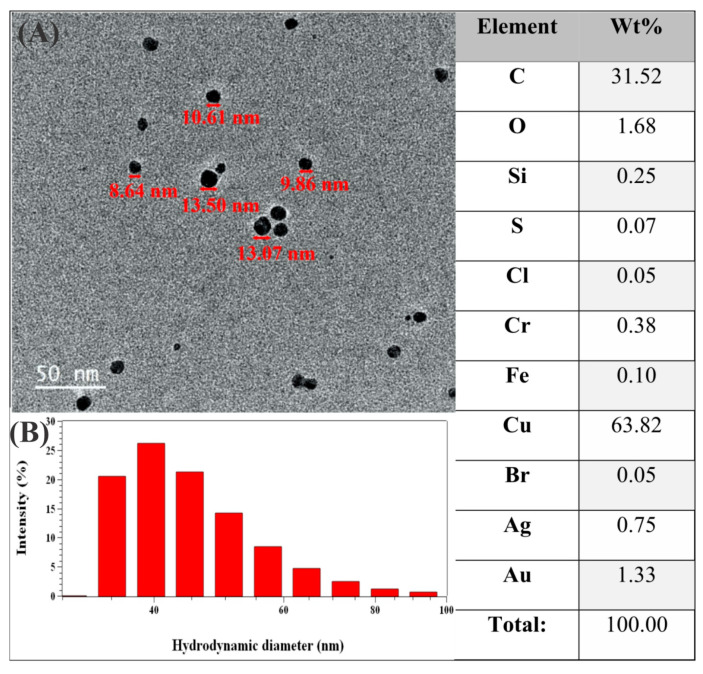
(**A**) Transmission electron microscopy of AgNPs (scale bar = 50 nm). (**B**) Dynamic light scattering of AgNPs dispersion. On the right is the table referring to the EDS analysis.

The NPs exhibited a spherical shape with low polydispersity. TEM images (Figure 2A), combined with EDS, confirmed the presence of silver with an average size of approximately 10 nm. DLS analyses, shown in Figure 2B, demonstrate that the AgNPs have an average size of 33.2 ± 2.1 nm. A monomodal distribution was observed, which aligned with the obtained PDI value of 0.398, indicating moderate polydispersity.PDI evaluates the size distribution of NPs; the closer the PDI is to 0, the more monodisperse the analyzed system is. Zeta potential analysis revealed that the colloids had an average surface charge of −51.8 ± 2.5 mV, which results in a stability of colloidal dispersion of AgNPs.

Complementary ICP analyses provided quantitative data on silver concentration, showing 0.01355 mg/mL of dispersed silver.

### 2.2. Chemical and Morphological Characterization of the BC-AgNP Nanocomposite

**Figure 3:**
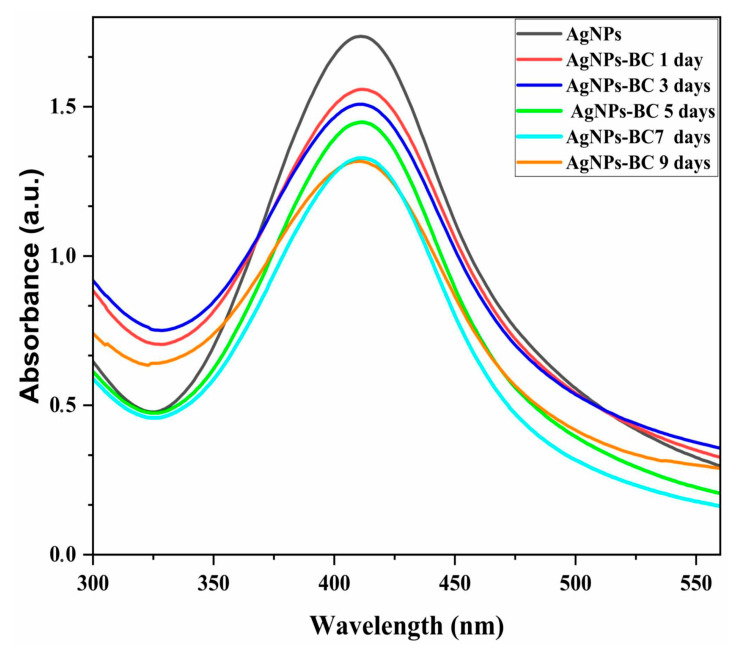
UV–Vis spectrum (black) of silver nanoparticles synthesized from tucumã plant extract, (red) BC–AgNP nanocomposite after 1 day of agitation, (dark blue) after 3 days of agitation, (green) after 5 days of agitation, (light blue) after 7 days of agitation, and (orange) after 9 days of agitation.

Figure 3 shows the UV–Vis spectra of the localized surface plasmon resonance (LSPR) band associated with AgNPs in colloidal dispersion, along with its gradual decrease upon incorporation into the BC matrix. The initial spectrum exhibited a maximum absorbance of 1.73 a.u. at approximately 410 nm, a region typically associated with spherical silver nanoparticles due to their characteristic LSPR [32].

The same figure also presents the spectral evolution during the incorporation process over a period of 7 days. As the reaction progressed, a progressive decline in absorbance intensity was observed, reaching 1.33 a.u. on the 7th day, suggesting continuous migration and immobilization of AgNPs onto the BC membrane. This reduction in the LSPR signal reflects the depletion of free nanoparticles in the dispersion, indicating successful incorporation into the polymeric network.

**Figure 4:**
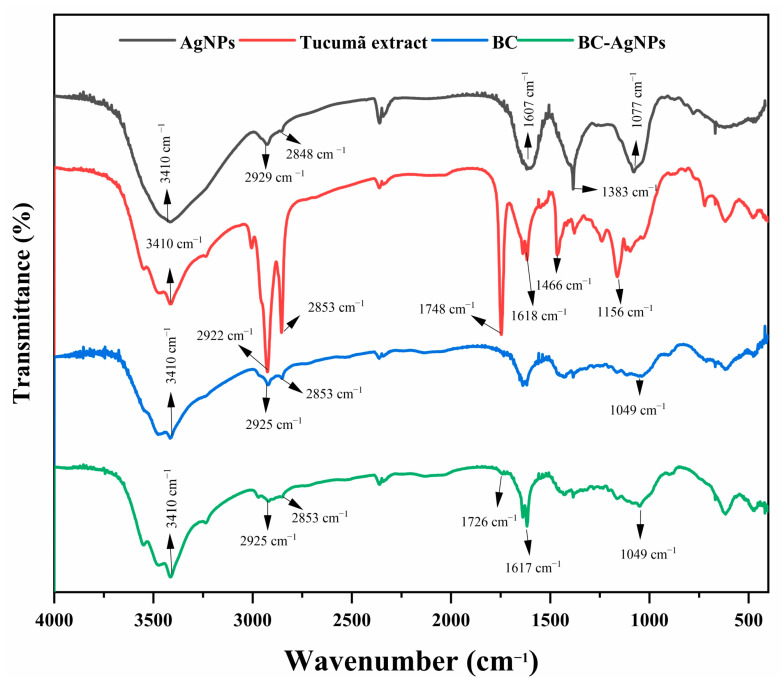
Fourier transform infrared spectrum—(black) AgNPs synthesized from tucumã extract, (red) tucumã plant extract only, (blue) bacterial cellulose, (green) BC–AgNP nanocomposite.

The FTIR spectra of BC, AgNPs, tucumã plant extract, and the BC–AgNP nanocomposite are shown in Figure 4. The presence of characteristic organic functional groups confirms the interaction between the components during nanocomposite formation. A prominent broadband at 3410 cm^−1^ is attributed to O–H stretching vibrations, commonly associated with hydroxyl groups from polysaccharides and phenolic compounds. The aliphatic C–H stretching bands appear at 2925 cm^−1^ and 2853 cm^−1^, indicating the presence of long-chain fatty acids and glycerides. The band at 1617 cm^−1^ is related to C=O stretching or aromatic C=C vibrations, suggesting the presence of carbonyl or aromatic groups, while the band at 1466 cm^−1^ corresponds to CH_2_ bending vibrations. The absorption at 1383 cm^−1^ is associated with CH_3_ symmetric bending, indicating methyl-containing groups in the plant extract. A distinct band at 1077 cm^−1^ can be attributed to C–O stretching vibrations of alcohols, carboxylic acids, esters, and ethers, or possibly C–N stretching in amines. The appearance and shifts of these bands occur in the BC–AgNP spectrum, particularly with the emergence of a new band at 1726 cm^−1^ [33].

**Figure 5:**
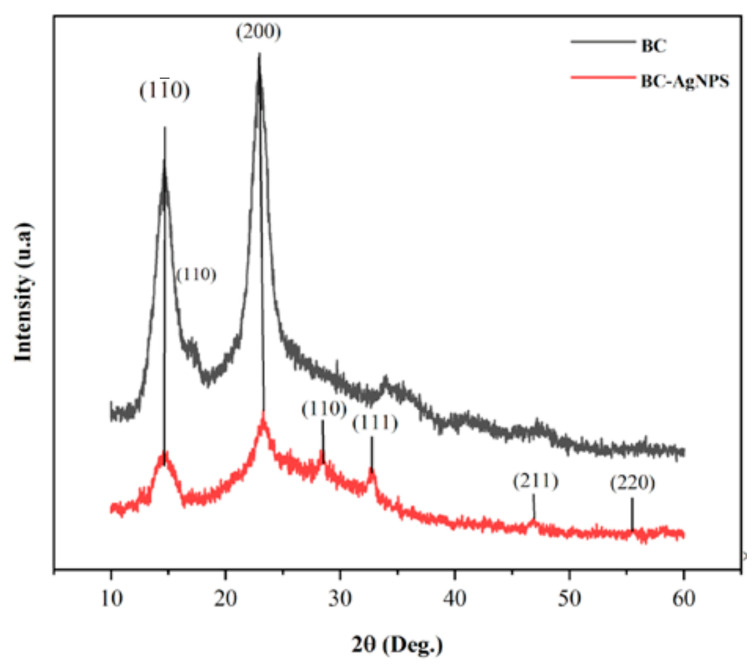
XRD spectrum—(black) pure BC and (red) BC–AgNP nanocomposite.

The XRD patterns of pure BC and the BC–AgNP nanocomposite are shown in Figure 5. The presence of strong peaks at 14.5°, 17.0°, and 22.8°, corresponding to the (1$\bar{1}$0), (110), and (200) planes, respectively, can be attributed to the crystalline phase of BC.

**Figure 6:**
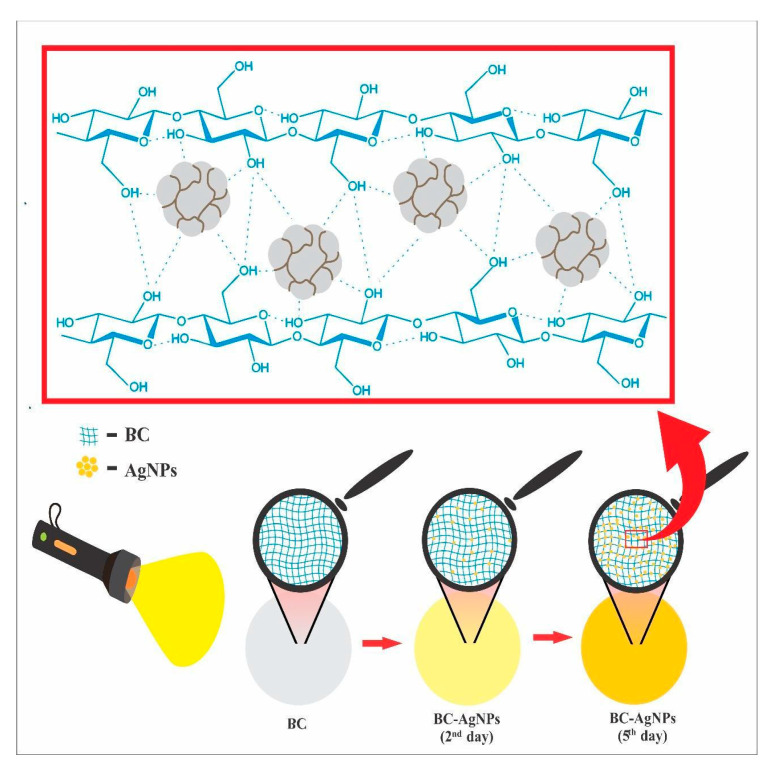
Schematic representation of the interaction between light and the BC–AgNP nanocomposite during the reaction period, illustrating the progressive immobilization of silver nanoparticles within the bacterial cellulose matrix.

**Figure 7:**
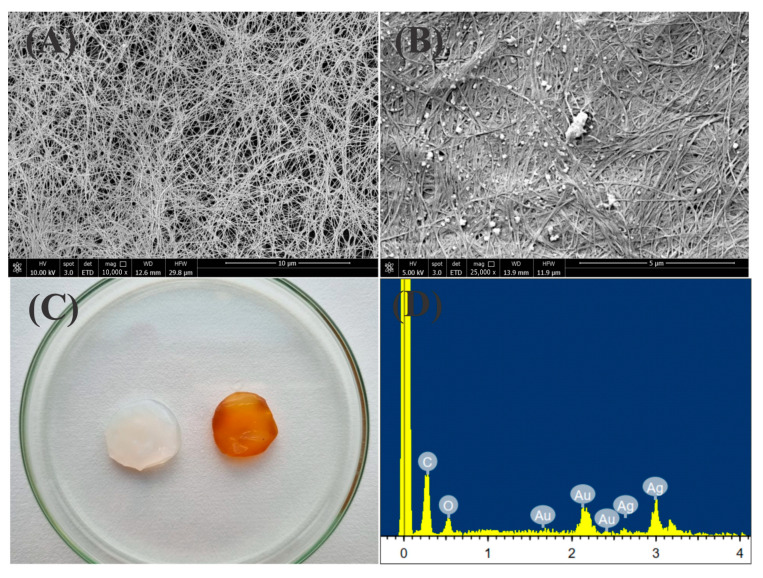
(**A**,**B**) Scanning electron microscopy images of BC and BC–AgNP, respectively. (**C**) Color change of the bacterial cellulose membrane, and (**D**) EDS analysis of the membrane with incorporated AgNPs.

As the reaction progresses, more AgNPs adhere to the surface of the BC, reaching a peak brown coloration on the 5th day (Figure 6 and Figure 7C). ICP analyses confirmed the presence and concentration of silver in BC at a concentration of 0.00215 mg/mL. Thus, the incorporation of AgNPs into the membrane was successful.

Figure 7A shows an SEM image, where a network of three-dimensional fibers with voids is identifiable, suggesting the successful removal of bacteria during BC purification. The gaps exposed in the interwoven cellulose fibers facilitate the immobilization of AgNPs. The SEM image of the freeze-dried BC–AgNP nanocomposite revealed significant differences in fiber structure, with AgNPs visible across the entire BC surface (Figure 7B). EDS analysis confirmed the presence of metallic silver (Figure 7D).

Table 1 shows the water absorption for pure BC and the BC–AgNP nanocomposite. Due to its porous structure, the BC–AgNP nanocomposite showed significantly lower swelling capacity compared to pure BC (*p* = 0.0037), indicating reduced water uptake.

### 2.3. Assay of Antimicrobial Activity

**Figure 8:**
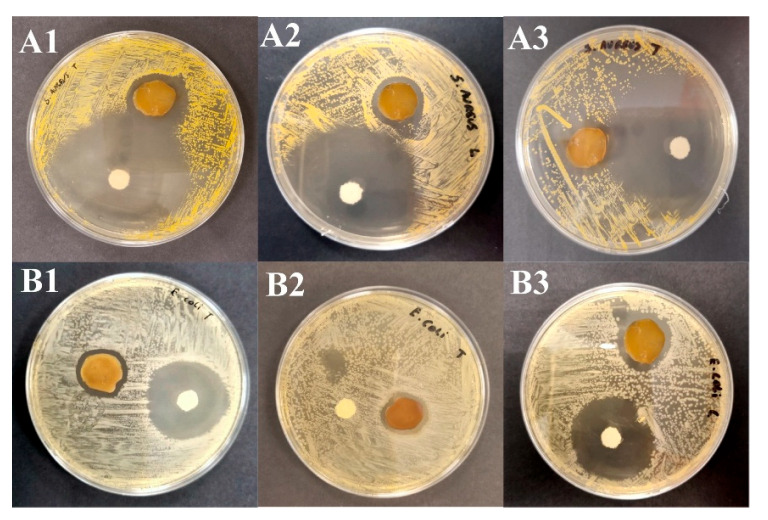
Assessment of antimicrobial activity using the disk diffusion method on agar plates against (**A1**–**A3**) *Staphylococcus aureus* and (**B1**–**B3**) *Escherichia coli*.

The disk diffusion method was used to evaluate the efficacy of the BC–AgNP nanocomposite against two bacterial species belonging to two groups: *E. coli* (Gram-negative) and *S. aureus* (Gram-positive). Figure 8 presents the primary results of the bactericidal assays. The BC–AgNP nanocomposite sample exhibited clear inhibition zones, measuring 6.5 ± 0.5 for *S. aureus* and 4.3 ± 0.3 mm for *E. coli*, respectively (Supplementary Material Table S1).

## 3. Discussion

The color change of the dispersion to light brown occurred progressively within the first 20 min of the reaction. This optical shift is attributed to the interaction of light with metallic nanostructures, inducing surface plasmon resonance (SPR), which leads to localized charge oscillations and enhancement of the electromagnetic field around the AgNPs [32,34].

EDS results revealed significant amounts of copper, gold, carbon, and oxygen. The presence of copper and gold in the EDS spectra is associated with the use of standard copper grids coated with gold, which are commonly employed in electron microscopy. The excitation of these substrates by the electron beam results in the detection of their characteristic signals [35,36,37]. The presence of carbon and oxygen suggests the adsorption of biomolecular functional groups from the plant extract onto the surface of the silver colloids, supporting their role in nanoparticle stabilization. Biomolecules in the plant extract, such as phenolic compounds and flavonoids, play dual roles in the reaction by reducing silver cations and stabilizing colloid growth [20,38,39].

The morphology and size of the synthesized NPs were evaluated using TEM and DLS, respectively (Figure 2). As a result, DLS typically reports larger particle sizes compared to TEM, due to the inclusion of the solvation shell and any adsorbed surface molecules. TEM measures the actual size of the nanoparticles, providing detailed images, while DLS measures the hydrodynamic size in solution, which includes the particle along with any solvent layer or adsorbed molecules. This difference generally causes the nanoparticle size obtained by DLS to be larger than that measured by TEM [40,41,42].

The polydispersity index (PDI) of the sample (0.398) indicates moderate polydispersity, consistent with values reported for AgNPs synthesized using plant extracts (0.3–0.5) [13,43,44]. This PDI value reflects a relatively narrow size distribution, though some degree of polydispersity is still present, as expected for green synthesis processes. Compared to other studies, Siakavella et al. [45] obtained a lower PDI (0.269), indicating greater homogeneity, whereas Lima et al. [46] reported higher values (0.523–0.689), reflecting greater variability. The PDI of the present sample suggests a good balance between colloidal stabilization and variability in the synthesis process.

Furthermore, the zeta potential of the AgNPs was measured at −51.8 ± 2.5 mV, which indicates excellent electrostatic stability. Zeta potential values above ±30 mV are generally considered stable, and values below −50 mV suggest strong repulsive forces between particles, effectively preventing aggregation. This result confirms the effective capping and stabilization of AgNPs by the phytochemicals present in the plant extract [47,48].

The silver content determined by ICP (0.01355 mg/mL) is lower than that reported by Singh and Mijakovic [49] (0.078 mg/mL), suggesting a reduced accumulation of AgNPs in the matrix. The silver concentration can be modulated through precursor (AgNO_3_) variation, allowing fine control of loading efficiency into the polymeric matrix. For wound dressing applications, the silver content should be optimized to minimize the risk of tissue toxicity and discoloration while maintaining antimicrobial efficacy [50,51,52].

The UV-Vis results shown in Figure 3 suggest a gradual decrease of nanomaterials in the colloidal system over time. This reduction suggests a progressive uptake of AgNPs by the membrane, reaching a saturation state after 7 days of agitation. In this reaction, electrostatic forces supported the incorporation of the nanostructures and their immobilization in the membrane pores, contributing to impregnation [53,54,55].

After the reaction between the plant extract and AgNO_3_ was completed, FTIR analysis revealed spectral changes that support the formation of silver nanoparticles. For instance, the bands at 2922 cm^−1^ and 2853 cm^−1^ observed in the plant extract shifted to 2929 cm^−1^ and 2848 cm^−1^ in the AgNPs spectrum, respectively, with a noticeable decrease in intensity. Additionally, a new and intensified band appeared at 1383 cm^−1^ in the AgNPs spectrum, possibly resulting from overlapping contributions or conformational changes of biomolecular groups during nanoparticle formation. These spectral variations indicate chemical changes in the plant-derived compounds responsible for the reduction and stabilization of AgNPs [56,57]. The shifts and intensity changes are consistent with previously reported transformations of phenolic structures, where interaction with Ag^+^ can promote electron donation and tautomeric conversion from enol to keto forms. The spectrum of the BC–AgNP nanocomposite retained all the characteristic bands of pure BC, with the exception of a new elongation band at 1726 cm^−1^. According to Fadakar [33], this band is indicative of a chemical interaction between Ag^+^ and functional groups in the bacterial cellulose, confirming the successful incorporation of AgNPs into the polymeric matrix.

The XRD pattern of the BC–AgNP nanocomposite reveals distinct peaks at 28.4°, 32.8°, 46.8°, and 55.6°, which correspond to the (110), (111), (211), and (220) planes of metallic silver (Ag^0^), respectively. These reflections are characteristic of the face-centered cubic (FCC) crystalline structure of silver, confirming the successful formation of AgNPs within the bacterial cellulose matrix. The presence of these peaks, absent in the pure BC pattern, indicates the incorporation of crystalline AgNPs without significantly altering the cellulose crystalline structure. Similar diffraction profiles have been reported in previous studies involving green synthesis of AgNPs [56,58,59]. Due to the low intensity of the AgNPs spectra, complementary techniques such as scanning electron microscopy (SEM), EDS, and ICP analyses were conducted to confirm the presence of silver.

The immobilization of these silver colloids in BC results in a color change in the membrane. This optical phenomenon arises from the interaction of light with the material, causing surface electrons to be excited to higher energy levels, known as surface plasmon resonance [32] (Figure 6). The incorporation of the nanostructures is facilitated by the interaction of the OH^−^ groups in the chemical structure of BC [57,60]. ICP analyses identified the presence and concentration of silver in BC at 0.0021 mg/mL. Thus, the incorporation of AgNPs into the membrane was successful.

Figure 7C displays the BC membrane with white coloration, whereas the BC–AgNP nanocomposite, after five days of immersion, shows brown coloration. These data suggest the successful incorporation of AgNPs into BC [56,57,59]. The BC–AgNP nanocomposite showed significantly lower swelling capacity compared to pure BC (*p* = 0.0037), indicating reduced water uptake. This behavior is attributed to a combination of reduced hydrophilicity, increased structural compactness, decreased availability of free hydroxyl groups, and altered cellulose–water interactions, making the membrane less permeable and less susceptible to moisture absorption [11,56,58]. The studies by Jenkhongkarn and Phisalaphong [58] evaluated the physical properties of bacterial cellulose (BC) films incorporated with AgNPs synthesized using different reducing methods. It was observed that the presence of AgNPs generally reduced the water absorption capacity of the films, indicating decreased hydrophilicity and increased structural compactness.

The bactericidal activity of the BC–AgNP nanocomposite is primarily attributed to the release of silver ions (Ag^+^), which interact with bacterial membranes, leading to increased permeability and structural disruption. Additionally, Ag^+^ promotes the generation of reactive oxygen species (ROS), inducing oxidative stress and damaging cellular components such as lipids, proteins, and DNA. The enhanced bactericidal effect observed against *S. aureus* may stem from differences in bacterial cell wall composition, as Gram-positive bacteria lack the outer membrane that hinders AgNPs penetration in Gram-negatives [12,61].

The silver concentrations in the dispersion (0.01355 mg/mL) and in the BC membrane (0.00215 mg/mL) are in line with values reported in previous studies and commercial formulations used for wound care, which aim to balance antimicrobial effectiveness with biocompatibility. This value corresponds to an incorporation of approximately 15.5% of the total silver initially present in the dispersion, confirming the efficiency of the loading process into the BC membrane [62,63,64,65]. These concentrations enable antimicrobial action without compromising cellular integrity, as demonstrated by recent studies evaluating the cytotoxicity of AgNPs at concentrations below 0.016 mg/mL [62,64]. Additionally, commercial products utilizing similar concentrations include dressings such as Acticoat^®^ and AQUACEL^®^ Ag, which are widely employed in wound healing due to their controlled release of silver ions [63].

Several studies have reported a correlation between silver content and its antimicrobial activity [66,67,68]. Sheng et al. [66] tested different concentrations of AgNO_3_ and identified the minimum concentrations for bacterial inhibition as 0.004 mg/mL for Gram-negative (*P. aeruginosa*) and 0.016 mg/mL for Gram-positive (*S. aureus*) bacteria. Recent studies suggest that the antibacterial effect of AgNPs can be influenced by various factors, including size, shape, surface charge, concentration, and ion release rate of the nanostructures. In a study conducted by Mohammad et al. [59], enhanced activity against Gram-positive bacteria was observed, while Fadakar et al. [57] demonstrated greater efficacy against Gram-negative bacteria (*E. coli*).

## 4. Materials and Methods

### 4.1. Material

Silver nitrate (99%) was sourced from Sigma-Aldrich (Darmstadt, Germany), sodium hydroxide from Labsynth (Diadema, São Paulo, Brazil), Mueller–Hinton agar, Nutrient Broth and Tryptic Soy Broth (TSB) from Condalab, Labotories Condo S.A. (Torrejón de Ardoz, Madrid, Spain), Leão green tea from the official Leão store (Vila Leopoldina, São Paulo, Brazil), BC from Probióticos Brasil (Santo André, São Paulo, Brazil), refined sugar from União (Teresina, Piauí, Brazil), and sodium hypochlorite solution (NaOH) from Êxodo ciêntifica (Alto da Mooca, São Paulo, Brazil).

### 4.2. Preparation of Bacterial Cellulose (BC)

BC was produced using green tea as the culture medium (20 g/L) supplemented with 100 g/L of sugar. A previously cultivated SCOBY (symbiotic culture of bacteria and yeast) was used as an inoculum, following the method of Treviño-Garza et al. [9]. The culture was incubated under static conditions at 30 °C for 7 days. The membranes formed on the surface were harvested, treated with 30% sodium hypochlorite for 48 h, and then rinsed in distilled water under agitation for 5 days with daily water changes until reaching neutral pH. Membranes were stored in distilled water until further use.

### 4.3. Production of Colloidal Dispersion of Silver Nanoparticles

Fresh *A. aculeatum* fruits were purchased at the municipal market Gesta Filho (−3°08′47.4″ S 58°26′57.0″ W) in Itacoatiara, Amazonas, Brazil. The mesocarp was separated, and 3 g were weighed and macerated with 5 mL of distilled water for 10 min. The mixture was then diluted with 195 mL of distilled water, allowed to rest for 24 h, and centrifuged using a Kasvi K14-400 centrifuge (Pinhais, Paraná, Brazil) for 10 min at 4000 rpm. The supernatant (liquid extract) was collected. For the synthesis of silver nanoparticles, 5 mL of the plant extract was mixed with 50 mL of a 0.1 mmol/L silver nitrate (AgNO_3_) solution, resulting in a total reaction volume of 55 mL. Immediately after mixing, the pH was adjusted to 9 using 0.05 mol/L sodium hydroxide (NaOH). The reaction mixture was maintained at 40 °C for 24 h under constant stirring at 400 rpm. Afterward, the solution was kept at room temperature (~30 °C) for at least 48 h without agitation. The synthesis protocol was adapted from Dos Santos et al. [21].

### 4.4. Characterization of AgNPs

#### 4.4.1. UV-Vis Spectroscopy

UV–Vis spectroscopy was performed using a UV–Vis spectrophotometer, Shimadzu UV-2600 (Nakagyo-ku, Kyoto, Japan). Distilled water was used as the blank in a spectral range of 190–750 nm to investigate the surface plasmon resonance band of AgNPs in the sample. After mixing the plant extract and silver nitrate, the pH value was adjusted to 9 and the temperature to 40 °C. The samples were monitored at 30 min, 1 h, 4 h, 8 h, 24 h, 48 h, and 72 h.

The formation of the AgNP–BC nanocomposite was monitored by tracking the surface plasmon resonance (SPR) band of the AgNPs using UV–Vis spectroscopy, following the incorporation of a bacterial cellulose (BC) membrane (1 cm diameter) into 55 mL of the AgNPs dispersion. At each predefined time interval (1 h; and 1, 3, 5, 7, 9, 11, 13, and 15 days), aliquots of the reaction medium were collected and transferred to quartz cuvettes for spectral analysis. These aliquots were taken directly from the dispersion containing the BC membrane to evaluate the reduction in SPR absorbance, which is indicative of the progressive incorporation of AgNPs into the BC matrix.

#### 4.4.2. Dynamic Light Scattering

Dynamic light scattering (DLS), polydispersity index (PDI), and zeta potential were conducted using equipment from SZ-100Z, Horiba instruments Inc. (Tokyo, Japan). Measurements were performed in triplicate at 25 °C, calibrated for 2 min before the start of the measurement with a detection angle of 90° and a refractive index of 1.33. The results obtained were averaged, and the standard deviation was calculated. The same instrument was used to analyze zeta potential. The same samples were used for measurements following the same standard of analysis quantity and calibration time. Average deviation and standard deviation were calculated.

#### 4.4.3. Transmission Electron Microscopy (TEM)

The morphology and dimensions of the silver nanoparticles (AgNPs) synthesized using the aqueous extract of *Astrocaryum aculeatum* (tucumã) were evaluated by transmission electron microscopy (TEM). The nanoparticle size distribution was analyzed using ImageJ software Version 1.54k, with 50 nanoparticles randomly selected and measured from the TEM images. The analyses were carried out using a JEOL JEM 2100-HT microscope operating at 200 kV with a LaB6 electron source (JEOL Ltd., Tokyo, Japan). For sample preparation, aliquots were placed onto 400-mesh copper grids coated with a thin carbon film (approximate hole size: 42 µm; PELCO^®^, Ted Pella Inc., Redding, CA, USA). To enhance contrast, UranyLess stain (Electron Microscopy Sciences, Hatfield, PA, USA) was applied. The samples were air-dried at ambient temperature, and imaging was performed after 24 h. Micrographs were captured using an UltraScan^®^ 4000 CCD digital camera (Oneview, Gatan, Pleasanton, CA, USA). Furthermore, elemental analysis was performed through energy-dispersive X-ray spectroscopy (EDS), integrated into the TEM system, to determine the chemical composition of the nanoparticles.

### 4.5. Incorporation of AgNPs into BC

The BC membranes and AgNPs dispersions were prepared separately. Purified BC membranes were cut into circular shapes with a diameter of 1 cm, immersed in the AgNPs dispersion, and placed under stirring with a magnetic stirrer for 7 days. The incorporation of AgNPs into BC was monitored using a UV–Vis spectrophotometer. Finally, the antimicrobial activity of the BC–AgNP nanocomposite was evaluated.

### 4.6. Characterization of the BC-AgNP Nanocomposite

#### 4.6.1. Morphological Study by Scanning Electron Microscopy (SEM)

To evaluate the morphology of pure BC and the BC–AgNP nanocomposite, the material was purified and lyophilized using the freeze-dryer Lyoquest −55 °C Plus Eco equipment (Telstar, Terrassa, Spain). Discs approximately 8 mm in diameter were cut and then coated with 10 nm of gold (Au). Then the morphology was evaluated using scanning electron microscopy (SEM) (QUANTA 650FEG—FEI Europe B.V. Company, Eindhoven, The Netherlands) and chemical elemental composition was determined with an integrated X-ray microanalysis system (EDS—energy dispersive spectrometer).

#### 4.6.2. Fourier Transform Infrared Spectroscopy (FTIR) Analysis

Fourier transform infrared spectroscopy (FTIR) was employed to identify the functional groups present in the plant extract, silver nanoparticles (AgNPs), bacterial cellulose (BC), and the BC–AgNP nanocomposite. The spectra were recorded using a Nicolet-NEXUS 610 spectrophotometer (Markham, ON, Canada) in the attenuated total reflection (ATR) mode, covering the spectral range from 4000 to 500 cm^−1^. Each sample was analyzed with 124 scans at a resolution of 4 cm^−1^.

#### 4.6.3. X-Ray Diffraction Analysis (XRD)

To identify the crystalline phase of pure BC and BC–AgNP membranes, X-ray diffraction (XRD) was performed using a diffractometer (Shimadzu XDR-600, Nakagyo-ku, Kyoto, Japan). Spectra were recorded at angles ranging from 10° to 60°.

#### 4.6.4. Degree of Swelling

Water absorption capacity was assessed by analyzing the swelling degree of pure BC and the BC–AgNP nanocomposite. The samples were cut and dried at room temperature (25 °C) and immersed in distilled water for 24 h. Equation (1) was used to evaluate water absorption.

(1) $$Water\;absorption\;\left(\%\right)=\frac{W2-W1}{W1}\times 100$$

where *W*1 and *W*2 are the weights of the samples in the dry condition (room temperature dry) and wet condition (g), respectively.

### 4.7. Inductively Coupled Plasma Analysis

The inductive coupling plasma (ICP) analysis–optical emission spectroscopy (ICP–OES) (ICPE-9000 spectrometer, Shimadzu, Japan) was used to determine the silver concentration in the BC-AgNP nanocomposite.

The membrane was frozen with liquid nitrogen and lyophilized. After the samples were submitted to a calcination process at 800 °C overnight, aqua regia was added in the crucibles to remove the silver (Ag) residues. Then, 13 mL of the diluted aqua regia was analyzed to determine the silver ions.

### 4.8. Antimicrobial Activity

The antimicrobial activity of the BC–AgNP nanocomposite was evaluated against Gram-negative *E. coli* (ATCC 43888) and Gram-positive *S. aureus* (ATCC 6538) using the disk diffusion method. Bacterial strains were activated in Tryptic Soy Broth (TSB) at 37 °C for 18–24 h and standardized to a 0.5 McFarland turbidity standard (~1.5 × 10^8^ CFU/mL). Then, 100 µL of each suspension was spread uniformly on nutrient agar plates using a sterile swab. Circular BC–AgNP nanocomposite membranes (1 cm diameter) were sterilized under UV radiation for 1 h and placed onto the agar surface. Amoxicillin (500 mg/5 mL) was used as a positive control, and pure BC membranes served as the negative control (Supplementary Material Figures S1 and S2). The plates were incubated at 37 °C for 24 h. After incubation, inhibition zone diameters were measured in millimeters using a digital caliper. All experiments were conducted in triplicate.

### 4.9. Statistical Analyses

The experimental data presented in this study are expressed as the mean ± standard deviation (SD) for n = 3. The analysis was conducted using the Student’s *t*-test, with the significance level (*p*-value) set at <0.05.

## 5. Conclusions

We developed a membrane with antibacterial properties, intended for future wound dressing applications, capable of maintaining ideal moisture and gas exchange conditions. UV–Vis, TEM, and DLS analyses demonstrated that favorable conditions were present during the synthesis of AgNPs from the tucumã plant extract, forming structures with an average size of 32.2 nm and demonstrating high stability, as confirmed by zeta potential measurements.

Data from SEM, energy-dispersive X-ray spectroscopy, XRD, and ICP analyses further validated the nanoparticle formation and incorporation process. Over the course of the reaction, there was a gradual incorporation of silver nanostructures into the BC membrane, resulting in a BC-AgNP nanocomposite with enhanced physicochemical properties. Concerning the FTIR results, the emergence of a stretching vibration band at 1617 cm^−1^ in the spectrum of the BC-AgNP nanocomposite suggests the successful incorporation of the nanoparticles into the polymer matrix. This incorporation occurs through interactions between the OH groups in the BC structure and the functional groups of the nanoparticles.

This work adopts a sustainable approach by leveraging the botanical richness of the Amazon and adding value to regional biodiversity. A BC–AgNP nanocomposite was produced through an optimized green synthesis route designed to minimize environmental impact. The results confirm that the synthesis method yielded a material with desirable properties for wound dressing applications.

The nanocomposite exhibited bactericidal activity against Gram-negative (*E. coli*) and Gram-positive (*S. aureus*) bacteria. In conclusion, the BC–AgNP nanocomposite exhibits promising potential as a wound dressing material, offering reduced production costs compared to conventional methods. However, additional tests, including biodegradation, toxicity, and process sustainability, should be conducted to qualify the nanocomposite for use as a wound dressing.

## Figures and Tables

**Table 1:** Percent water absorption over 24 h in pure BC and the BC–AgNP nanocomposite.

Samples	Dry Weight (g)	Wet Weight (g)	Average Water Absorption (%)
BC	0.012 ± 0.0008	0.105 ± 0.006	777
BC–AgNP	0.018 ± 0.0007	0.118 ± 0.0015	556

## Data Availability

The raw data supporting the conclusions of this article will be made available by the authors on request.

## Supplementary Materials

The following supporting information can be downloaded at https://www.mdpi.com/article/10.3390/ph18060799/s1, Figure S1: Bactericidal inhibition assay using *Escherichia coli* as a model; Figure S2. Bactericidal inhibition assay using *Staphylococcus aureus* as a model; Table S1: Inhibition zone diameters (in mm) against *Staphylococcus aureus* and *Escherichia coli* for the BC–AgNPs nanocomposite.

## Abbreviations

AgNO_3_	Silver nitrate
AgNPs	Silver nanoparticles
BC	Bacterial cellulose
BC-AgNP	Nanocomposite of silver nanoparticles and bacterial cellulose
DLS	Dynamic light scattering
EDS	Energy dispersive spectrometer
FTIR	Fourier transform infrared spectroscopy
ICP	Inductively coupled plasma analysis
NaOH	Sodium hypochlorite
NPs	Nanoparticles
OH^−^	Hydroxyl ion
PDI	Polydispersity index
SEM	Scanning electron microscopy
TEM	Transmission electron microscopy
UV-Vis	Ultraviolet–visible microscopy
XRD	X-ray diffraction analysis
