# Supplementary material for: Biotechnological Utilization of Amazonian Fruit: Development of Active Nanocomposites from Bacterial Cellulose and Silver Nanoparticles Based on Astrocaryum aculeatum (Tucumã) Extract
Source: Pharmaceuticals (Basel). 2025 May 26;18(6):799. doi: 10.3390/ph18060799 (PMC12195901; doi:10.3390/ph18060799)
Supplement: Supplementary file 1 [file pharmaceuticals-18-00799-s001.zip › pharmaceuticals-3527044-supplementary.pdf]

1. Supplementary Materials

Figure S1. Bactericidal Inhibition Assay Using *Escherichia coli* as a Model. Plate (A) represents the bacterial culture (negative control), Plate (B) contains only bacterial cellulose (negative control), and Plate (C) contains 5 mg/mL of Amoxicillin (positive control).

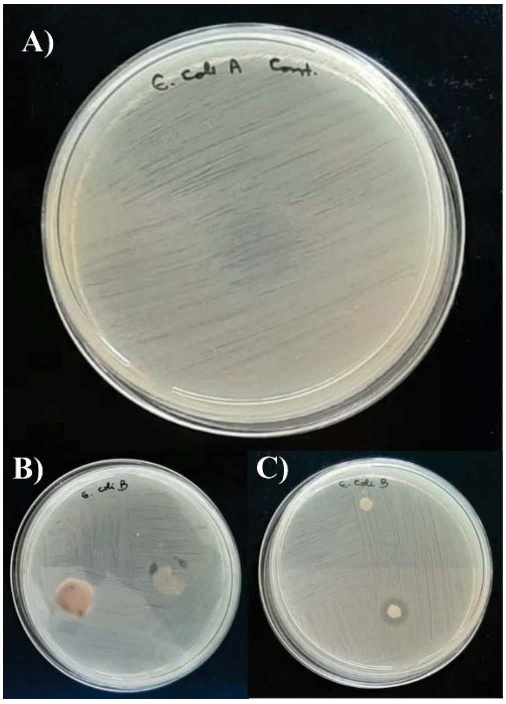

Figure S2. Bactericidal Inhibition Assay Using *Staphylococcus aureus* as a Model. Plate (A) represents the bacterial culture (negative control), Plate (B) contains only bacterial cellulose (negative control), and Plate (C) contains 5 mg/mL of Amoxicillin (positive control).

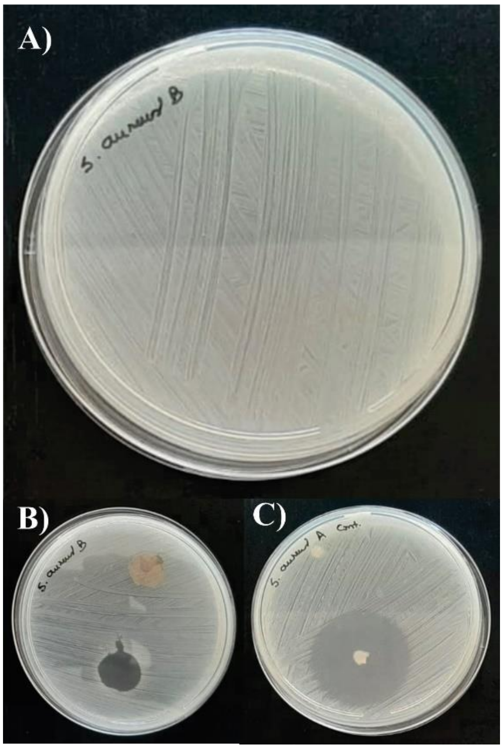

Table S1. Inhibition zone diameters (in mm) against *Staphylococcus aureus* and *Escherichia coli* for the BC–AgNPs nanocomposite. Values represent the diameter of inhibition halos for three replicates (N1–N3), with calculated means and standard deviations.

**Sample**	***S. aureus* (mm)**	***E. coli* (mm)**
**N1**	6.2	4.4
**N2**	7.1	4.6
**N3**	6.5	4.0
**Mean**	**6.5**	**4.4**
**Standard Deviation**	**0.46**	**0.31**
